# Decoupling Heat and Electrical Conduction in Bilayer Graphene Through Wrinkling‐Induced Phonon Hybridization

**DOI:** 10.1002/advs.202516792

**Published:** 2025-10-30

**Authors:** Aoran Fan, Wenlong Dong, Xiaolong Yang, Ya Hu, Yufeng Zhang, Wu Li, Jun Lyu, Luqi Liu, Xing Zhang, Lin Yang

**Affiliations:** ^1^ Department of Engineering Mechanics Tsinghua University Beijing 100084 China; ^2^ CAS Key Laboratory of Nanosystem and Hierarchical Fabrication National Center for Nanoscience and Technology Beijing 100190 China; ^3^ College of Physics, and Center of Quantum Materials and Devices Chongqing University Chongqing 401331 China; ^4^ Eastern Institute for Advanced Study Eastern Institute of Technology Ningbo 315200 China; ^5^ Department of Advanced Manufacturing and Robotics College of Engineering Peking University Beijing 100871 China; ^6^ National Key Laboratory of Advanced Micro and Nano Manufacture Technology Peking University Beijing 100871 China

**Keywords:** anisotropic electrical conduction, anisotropic heat conduction, phonon hybridization, wrinkled graphene

## Abstract

2D materials like graphene are renowned for their exceptional thermal and electrical properties, yet their performance can be significantly altered by structural irregularities such as wrinkles. While previous studies have reported modulation of thermal conductivity (*κ*) and electrical resistance (*R*) in wrinkled graphene, the results are often inconsistent or even contradictory, primarily due to challenges in experimentally disentangling geometric distortion from lattice strain. Here, a nearly zero‐strain wrinkling strategy is introduced for bilayer graphene (BLG) and uncover a strikingly inverse anisotropic relationship: thermal conductivity perpendicular to the wrinkles (*κ*_⊥_) is lower than that parallel to the wrinkles (*κ*_∥_), whereas electrical resistance exhibits the opposite trend, with *R*_⊥_ lower than *R*_∥_, highlighting the decoupling of thermal and electrical transport in wrinkled graphene. Atomistic simulations reveal that this behavior arises from phonon mode hybridization induced by out‐of‐plane geometric perturbations, which decelerates heat‐carrying phonon modes across the wrinkles and modifies electron–phonon scattering, thereby governing both the thermal conductivity and phonon‐limited electrical resistance. This work advances the understanding of energy carrier transport in wrinkled 2D materials and provides new insights into directionally modulating heat and charge flow in advanced electronic devices.

## Graphical abstract

Measurements of wrinkled bilayer graphene reveal a strikingly inverse anisotropic relationship: thermal conductivity perpendicular to the wrinkles is lower than that parallel to the wrinkles, whereas electrical conductivity exhibits the opposite trend, highlighting the decoupling of thermal and electrical transport in wrinkled graphene. Atomistic simulations reveal that this behavior arises from phonon mode hybridization induced by out‐of‐plane geometric perturbations.

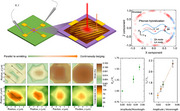

## 1 Introduction

Wrinkling is a ubiquitous phenomenon in 2D van der Waals materials.^[^ ^1^, ^2^, ^3^ ^]^ In large‐scale growth graphene growth on metallic substrates and subsequent transfer process, high densities of wrinkles are commonly observed due to thermal expansion mismatch^[^ ^4^ ^]^ or external mechanical loading.^[^ ^5^ ^]^ These wrinkles significantly influence graphene's properties, leading to local charge accumulation,^[^ ^6^ ^]^ and modulation of electrical,^[^ ^7^, ^8^ ^]^ thermal,^[^ ^9^, ^10^, ^11^, ^12^ ^]^ chemical^[^ ^13^ ^]^ as well as mechanical^[^ ^14^ ^]^ properties. Moreover, the resulting structural irregularities pose significant challenges for device reproducibility and reliability.^[^ ^15^, ^16^ ^]^ Despite significant strides in comprehending the effects of wrinkling, disentangling the intertwined influences of lattice strain and geometrical distortion on thermal and electrical transport remains unresolved.

Unlike bulk materials, where compressive strain often enhances thermal conductivity,^[^ ^17^ ^]^ 2D atomic layers such as graphene tend to buckle or wrinkle under compression to release stored elastic energy.^[^ ^18^ ^]^ Theoretical studies indicate that the structure change will significantly affect the transport properties of phonons,^[^ ^19^, ^20^, ^21^ ^]^ and further influence thermal transport properties. Molecular dynamics (MD) simulations have shown that the thermal conductivity of wrinkled graphene is lower along perpendicular direction (*κ*_⊥_) to the wrinkles than parallel (*κ*_∥_) to them,^[^ ^22^, ^23^ ^]^ yet the mechanisms are attributed to either bond length elongation due to strain^[^ ^22^ ^]^ or phonon localization caused by geometrical disturbance.^[^ ^23^ ^]^ These discrepancies call for direct experimental validation. Raman thermometry measurements have reported up to a ≈27% reduction in *κ* for wrinkled graphene compared to flat counterparts.^[^ ^24^ ^]^ However, the co‐existence of cracks and wrinkles during sample preparation complicated the data interpretation.^[^ ^24^ ^]^ In contrast, measurements on wrinkled molybdenum disulfide (MoS_2_) thin films found no significant change in *κ* compared to smooth samples, adding to the ongoing debate.^[^ ^25^ ^]^

Similarly, electrical resistance, *R*, in wrinkled graphene also shows conflicting trends. Some studies report increased *R* in both perpendicular, *R*_⊥_, and parallel, *R*_∥_, directions due to strain‐degraded carrier mobility,^[^ ^7^ ^]^ while others observe a decrease in *R* with increasing surface roughness, attributed to enhanced interlayer tunneling pathways within the wrinkled architecture.^[^ ^8^ ^]^ These inconsistencies highlight the complex interplay between mechanical strain and geometric distortion, both of which can critically impact electronic transport.

Clarifying these effects is essential for thermoelectric^[^ ^26^, ^27^ ^]^ and wearable device applications,^[^ ^28^, ^29^ ^]^ where decoupled control of heat and charge transport is highly desirable. Yet, inducing and probing well‐defined wrinkle geometries in suspended 2D materials—free from substrate interference—remains technically challenging. Existing methods, such as thermal expansion mismatch^[^ ^30^ ^]^ or push‐to‐shear strategy,^[^ ^31^ ^]^ often introduce significant tensile or shear strains (>1%), making it difficult to isolate pure geometric effects.^[^ ^30^, ^31^ ^]^ To date, no experimental study has simultaneously measured both *κ* and *σ* in the same wrinkled graphene sample, leaving a key gap in our understanding of wrinkle‐governed transport phenomena.

In this work, we developed a nearly zero‐strain strategy to prepare suspended bilayer wrinkled graphene, enabling direct measurement of both *κ* and *R* along directions perpendicular and parallel to the wrinkles. Remarkably, we observed an inverse anisotropic relationship: *κ*_⊥_ is lower than *κ*_∥_, while *R*_⊥_ is lower than *R*_∥_, demonstrating that wrinkling can effectively decouple thermal and electrical transport in graphene. Through coupling first‐principles calculations with MD simulation, we revealed that the anisotropic *κ* originates from the disappearance of a favored phonon polarization perpendicular to the wrinkling direction, limiting heat transport. In contrast, the anisotropic *R* arises from direction‐dependent phonon hybridization effects induced by the wrinkled geometry, which selectively modulates electron‐phonon scattering depending on the current direction. Together, these findings provide atomistic insight into how controlled wrinkling can be harnessed to engineer direction‐specific transport properties in 2D materials.

## 2 Results and Discussion

### 2.1 Wrinkled Graphene with Controlled Morphology

**Figure 1:**
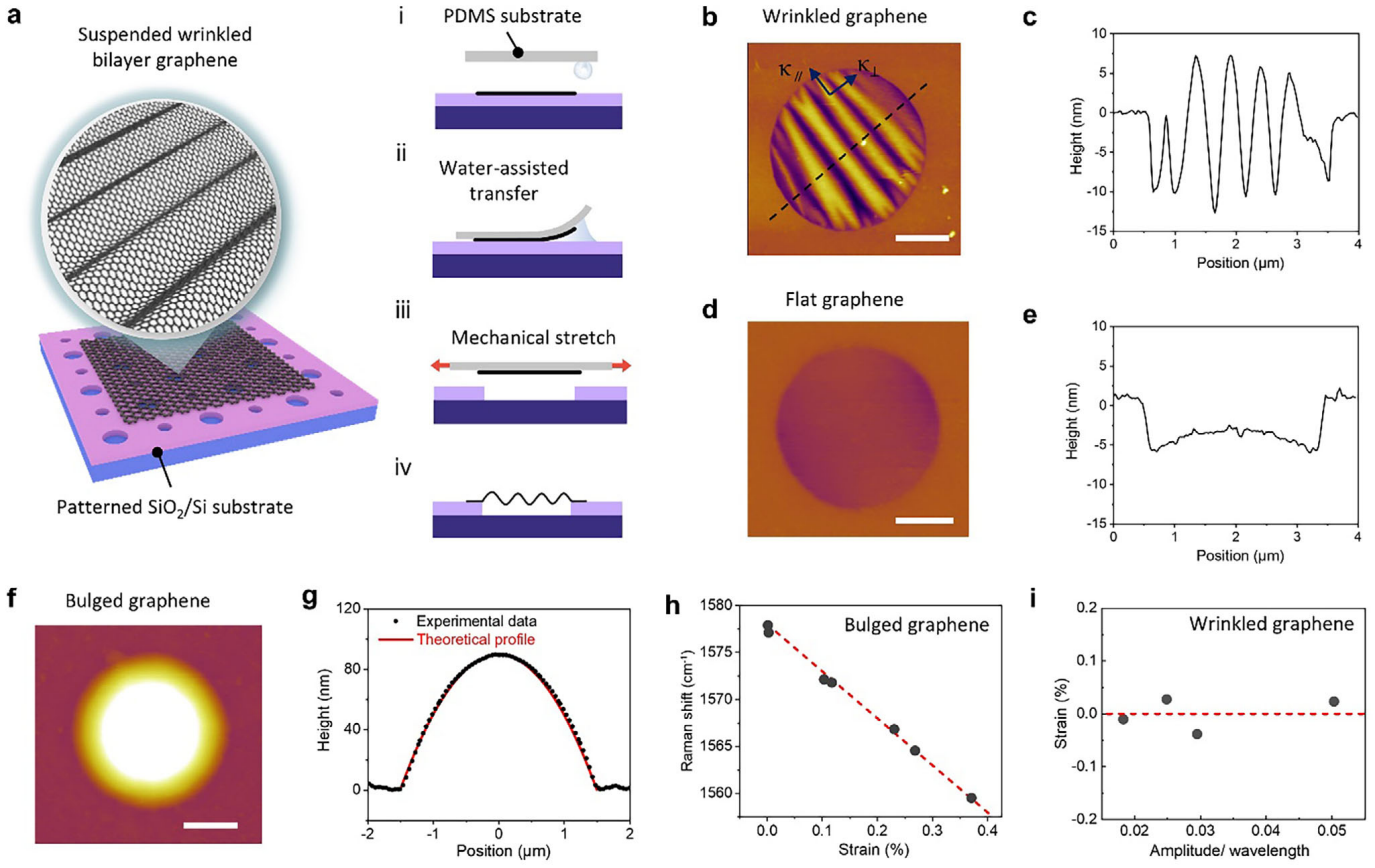
Wrinkled bilayer graphene sample preparation. a) Left panel: schematic diagram showing the wrinkled bilayer graphene (BLG) suspended on patterned SiO_2_/Si substrate. Right panel: sample preparation process flow: i‐ii. water‐assisted transfer, iii. mechanical stretch, and iv. PDMS release. b) AFM scanning image of a wrinkled BLG sample and c) its cross‐section profile. d) AFM scanning image of a flat BLG sample and e) its cross‐section profile. f) AFM scanning image of a bulged BLG sample and g) its cross‐section profile, where the red line is the morphological solution based on a thin membrane theoretical model. h) G band peak position shift for a flat BLG sample in response to loading strain via bulging. i) Residual strain within the wrinkled BLG of different amplitude/wavelength ratios. Scale bar in b, d, and f): 1 µm.

We have developed a nearly zero‐strain wrinkling technique that enables the formation of periodic patterns in suspended bilayer graphene (BLG). **Figure** **1**a illustrates the sample preparation process used to achieve suspended graphene wrinkles with tunable wavelengths and amplitudes (see details in Experimental Section). The procedure begins by exfoliating AB‐stacked BLG sheets onto a SiO_2_/Si substrate, followed by their transfer onto a polydimethylsiloxane (PDMS) substrate via a water‐assisted method.^[^ ^32^, ^33^ ^]^ Next, the BLG is transferred onto a SiO_2_/Si substrate featuring patterned holes. During this transfer, the mismatched Young's modulus between PDMS and graphene inevitably introduces residual strain in the graphene sheet. Upon removal of the PDMS substrate, the release of this residual strain prompts the formation of periodic wrinkles in the suspended regions. Importantly, this wrinkling process results in geometrical distortion without imparting lattice strain to the BLG, as elaborated later.

Figure 1b,c shows AFM images and cross‐sectional profiles of a wrinkled BLG sample. By carefully controlling the stretched degrees of the PDMS substrate, we can modulate the wavelengths (*λ*) and amplitudes (*ω*) of the wrinkled BLG. In this work, four wrinkled BLG samples were prepared and measured, with wavelength ranging from ≈391 to 669 nm, and amplitude from ≈9.4 to 19.8 nm (Figure S2, Supporting Information). To quantitatively evaluate the residual strain stored in the BLG sheets, we employed the flat BLG (Figure 1d,e) as a reference for biaxial stretch using a bulging device^[^ ^34^ ^]^ (Figure 1f,g). By introducing gas diffusion to create a pressure difference between the sealed cavity shown in Figure 1a and the external environment, the suspended BLG is pushed upward, undergoing continuous strain loading via a controlled expansion process. Based on the central cross‐sectional profile of the bulged BLG (Figure 1g), the loading strain at the bubble center point can be determined^[^ ^35^, ^36^ ^]^ (Supplementary Note 1, Supporting Information).

The Raman spectrum serves as a powerful tool for quantifying strain in deformed graphene sheets.^[^ ^37^, ^38^ ^]^ Here, we recorded the Raman signal from a BLG blister under varying bulging states, and plotted the G band peak positions against the corresponding strain values (Figure 1h). The results reveal a linear redshift of the G peak with increasing strain, yielding a Gruneisen parameter (*γ*) of 1.59, consistent with prior studies on strained graphene.^[^ ^38^, ^39^ ^]^ Based on the measured G band shift and the Gruneisen parameter, we can accurately determine the strain within the wrinkled BLG (Supplementary Note 2, Supporting Information). As shown in Figure 1i, the strain approaches nearly zero (less than 0.05%) for all four BLG samples exhibiting periodical wrinkles. Consequently, we are able to concurrently determine both the geometric parameters and lattice strain, thereby facilitating a comprehensive examination of phonon transport dynamics in wrinkled BLG.

### 2.2 Anisotropic κ Due to Wrinkling

**Figure 2:**
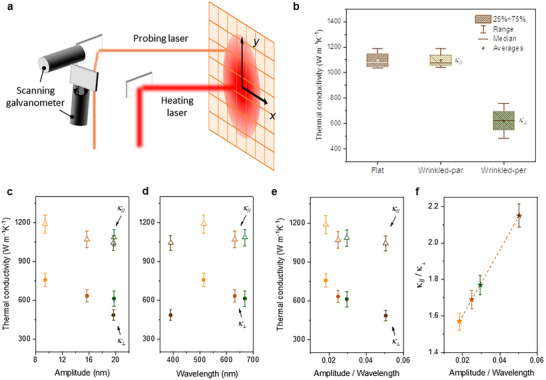
Anisotropic *κ* in winkled graphene. a) Schematic drawing showing the experimental system for anisotropic thermal conductivity characterization. b) Measured thermal conductivity for flat and wrinkled BLG. The box in the range 25–75% represents the middle 50% of the measured data, from lower quartile (25th percentile) to upper quartile (75th percentile). The range represents minimum to maximum value of the measured data. Median is the 50th percentile, and average is the mean value of the data. Measured thermal conductivity of wrinkled BLG versus c) amplitude, d) wavelength, and e) amplitude/wavelength, where *κ*_∥_ and *κ*_⊥_ represent thermal conductivity parallel and perpendicular to wrinkles, respectively. f) The ratio of *κ*_∥_/*κ*_⊥_ is plotted versus the amplitude/wavelength of wrinkles. Error bars represent the measurement uncertainty as analyzed in Supplementary Note 4 (Supporting Information).

We measured the thermal conductivity perpendicular, *κ*_⊥_, and parallel, *κ*_∥_, to the wrinkling direction using the two‐step dual‐wavelength Raman technique.^[^ ^40^, ^41^ ^]^ In this method, a high‐power heating laser heats the sample, and a probing laser with a different wavelength and lower power measures the Raman characteristic peak of the sample, whose position varies linearly with temperature rise (**Figure** **2**a). Based on the measured temperature distribution, heating power and geometric parameters, we extracted the thermal conductivity of the BLG sample along different directions (Experimental Section, Supplementary Note 3, Supporting Information). ^[^ ^40^, ^41^ ^]^

In this study, we prepared and measured four flat and four wrinkled BLG samples. Figure 2b displays the measured *κ* of the flat sample, along with *κ*_⊥_ and *κ*_∥_ for the wrinkled BLG. Interestingly, *κ*_∥_ of wrinkled BLG remains nearly identical to that of the flat counterpart, but *κ*_⊥_ decreases by 43% compared to flat samples. This finding is markedly different from previous MD modeling results, where both *κ*_⊥_ and *κ*_∥_ exhibited a decreasing trend due to wrinkling.^[^ ^23^ ^]^ To further examine the wrinkling effects on thermal transport, we plot the measured *κ*_⊥_ and *κ*_∥_ with respect to wavelength and amplitude, respectively, as shown in Figure 2c,d. While *κ*_∥_ remains nearly constant despite variations in the wrinkling configuration, no clear trends could be observed for *κ*_⊥_. Notably, even for samples with similar wrinkle amplitude *λ* ≈19 nm, the measured *κ*_⊥_ of different samples could be quite different (Figure 2c), suggesting that both *λ* and *ω* are critical in evaluating the wrinkling effects.^[^ ^18^ ^]^

As such, we plot the measured thermal conductivity as a function of the ratio of amplitude over wavelength, *λ*/*ω* (Figure 2e). A larger *λ*/*ω* ratio indicates graphene wrinkles with larger amplitude and smaller wavelengths, representing an increased level of wrinkling. Clearer trends emerged from Figure 2e: *κ*_∥_ initially decreases at small *λ*/*ω* ratio and then stabilizes as *λ*/*ω* further increases. In contrast, *κ*_⊥_ demonstrates a continuously decreasing trend as *λ*/*ω* increases. Notably, the decrease in *κ*_⊥_ by 36% is approximately three times greater than that of *κ*_∥_ (≈12%). Consequently, the thermal conductivity anisotropy, defined as the raito *κ*_∥_/*κ*_⊥_, consistently increases with *λ*/*ω*, reaching a value of ≈2.15 at a *λ*/*ω* ratio of 0.05. This represents the first experimental demonstration of anisotropic heat conduction induced by wrinkling, pointing toward a fundamentally new avenue for precise control over heat transfer directionality.

### 2.3 Eliminating Wrinkles Through Bulging

**Figure 3:**
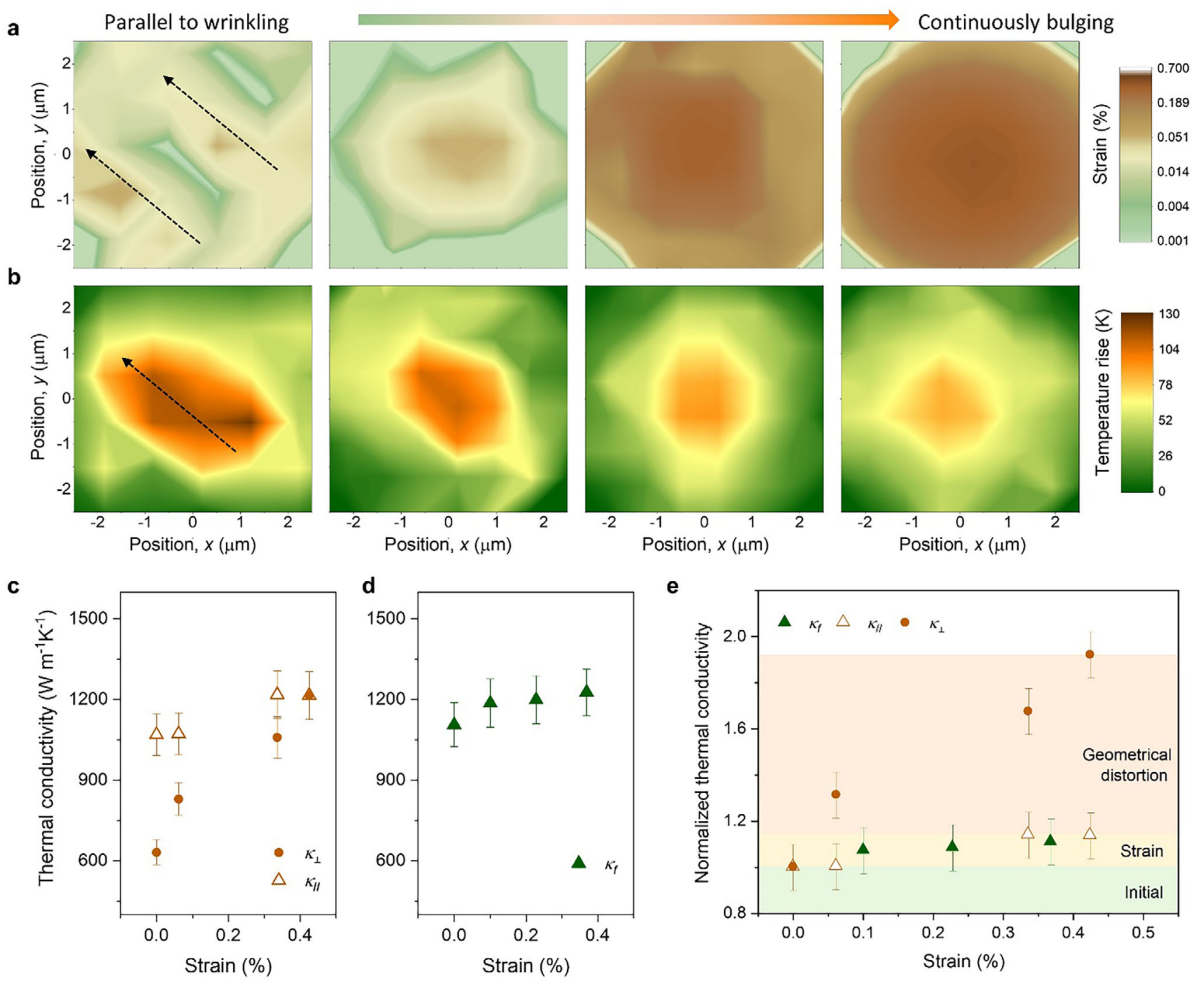
Eliminating wrinkles through bulging. Measured a) strain and b) temperature rise distribution of wrinkled bilayer graphene under different stages of bulging. Measured thermal conductivity versus strain for c) wrinkled and d) flat BLG. e) Normalized thermal conductivities plotted as a function of strain loading, where the normalization parameter is the thermal conductivity of each sample without pressure loading.

To further elucidate the wrinkling effects, we pressurized the same wrinkled BLG sample to form a bulge,^[^ ^34^ ^]^ continuously characterizing the strain distribution and thermal conductivity as the wrinkles diminished. **Figure** **3**a illustrates the strain evolution of the BLG sample under different stages of bulging. Utilizing the novel sample preparation technique described earlier, we again confirmed the very low residual strain (<0.05%) in the wrinkled BLG, allowing us to isolate the effects of geometric distortion on thermal transport. As the bulging progresses, the pressure difference between the sealed cavity and the external environment (Figure 1a) exerts a continuous strain load on the suspended BLG,^[^ ^35^ ^]^ leading to wrinkle elimination and an increase in elastic strain, with the strain at the center reaching ≈0.43% at the final stage.

**Figure 4:**
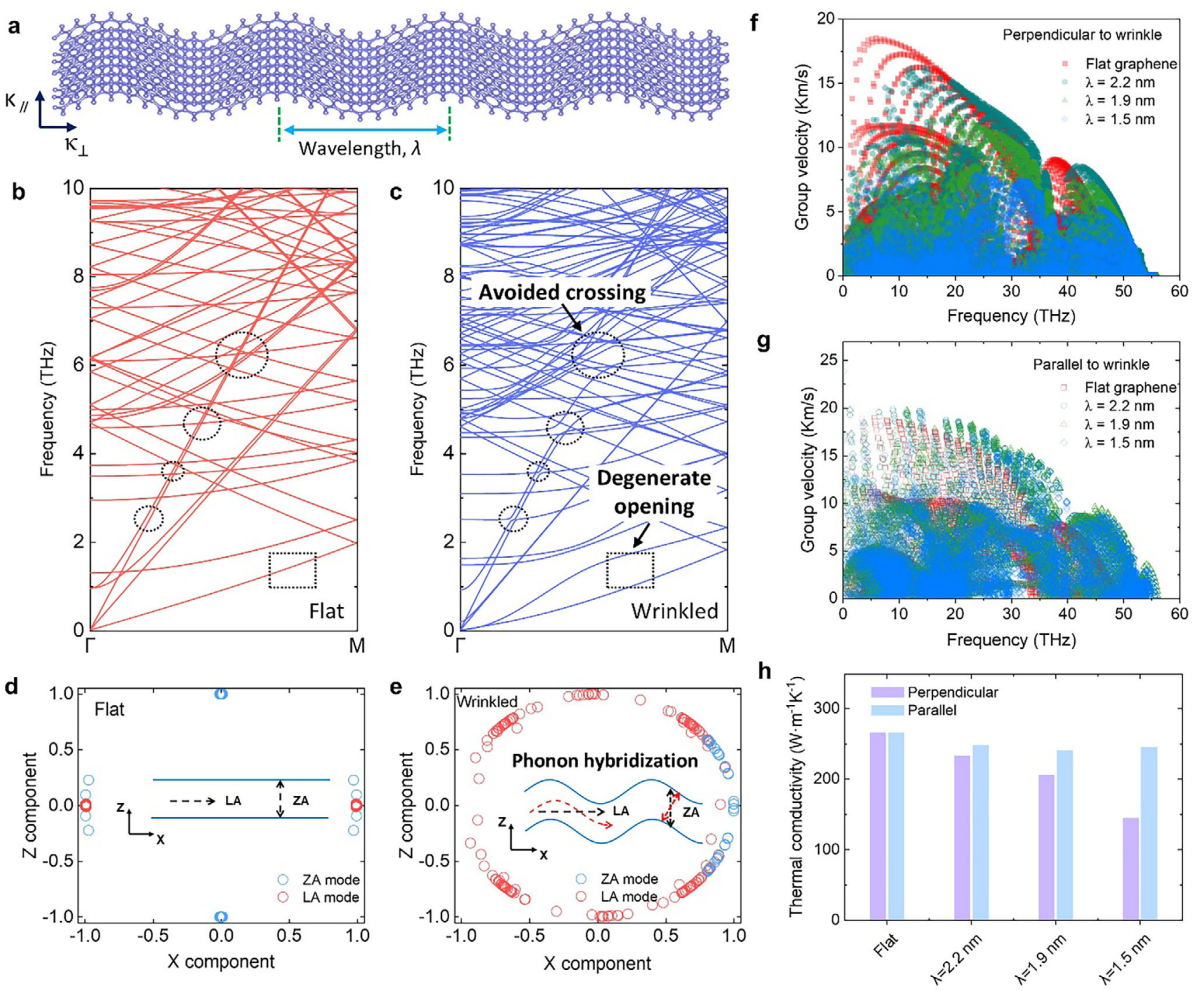
Phonon band avoided crossing and degenerate opening in wrinkled graphene. a) Schematic illustration of compression‐induced periodic wrinkles with a wavelength λ in a suspended BLG sheet. Zoom‐in plots of the calculated phonon dispersions for b) flat and c) wrinkled samples with λ of 1.9 nm. Avoided‐crossing behavior is marked by the dashed circles, and the degenerate phonon opening is highlighted by the dashed rectangles. *X–Z* components of the ZA (LA)‐mode polarization vectors around 1 (5) THz for d) flat and e) wrinkled (*λ* =1.9 nm) BLG. Calculated mode‐dependent phonon group velocities for different wrinkled samples f) perpendicular and g) parallel to the wrinkling direction, where the results for the flat sample are also plotted for comparison. h) Calculated room‐temperature *κ* perpendicular and parallel to the wrinkling direction using the NEMD simulations, the wrinkled BLG are with wrinkling wavelength of 2.2, 1.9, and 1.5 nm, respectively.

The disappearance of wrinkles is also evident in the temperature distribution measurements. **Figure** **4**b displays an elliptical temperature profile for the wrinkled BLG, with the long axis aligned along the direction of wrinkling, indicating a larger *κ*_∥_ compared to *κ*_⊥_. As the wrinkles diminished, the temperature distribution gradually evolves into a circular shape for the bulged BLG. Figure 3c plots the measured *κ*_∥_ and *κ*_⊥_ for the same BLG sample under different bulging states. *κ*_∥_ of the wrinkled BLG exhibits a slight increase with increasing strain, resembling the behavior observed in a flat BLG under continuous bulging (Figure 3d). The modest increase in *κ*_∥_ is consistent with previously modeled results of graphene under moderate tensile strain.^[^ ^9^, ^10^ ^]^ In contrast, even a small increase in tensile strain results in a significant enhancement in *κ*_⊥_, which becomes nearly identical to *κ*_∥_ as the strain reaches 0.43%, thereby restoring the isotropic heat conduction characteristics of pristine BLG.

Figure 3e further plots the variations in normalized thermal conductivities as a function of strain loading, where the normalization parameter is the thermal conductivity of each sample without pressure loading. Under similar strain loading conditions, the change in *κ_f_* and *κ*_∥_ is <14%, while the change in *κ*_⊥_ exceeds 92%. This significant difference underscores that the variation in *κ*_⊥_ is predominantly influenced by geometric distortions rather than by strain effects. Thus, by progressively bulging the wrinkled BLG sample and comparing the measured thermal conductivity, we experimentally validate that the observed anisotropic heat transport is indeed a consequence of geometric distortions caused by wrinkling.

### 2.4 Wrinkling‐Induced Phonon Mode Hybridization

To unravel the microscopic mechanism behind the suppressed *κ*_⊥_, we conducted lattice dynamics calculations combined with MD simulations on wrinkled BLG (Experimental Section). Figure 4a illustrates a schematic of a suspended bilayer graphene sheet with wrinkles formed to relieve in‐plane compressive strain. We constructed three different wrinkled BLG through controlling the compressive strain, as characterized by the height profiles (Figure S12, Supporting Information). We calculated the phonon dispersions of wrinkled BLG to show the wrinkling effects on phonon transport (Experimental Section). Note that wrinkling disrupts the periodicity of the graphene lattice, leading to the formation of a large quasi‐supercell in real space with a correspondingly reduced Brillouin zone. This disruption causes a phonon band folding effect,^[^ ^42^, ^43^ ^]^ making direct comparisons with the phonon spectrum of flat BLG in the primitive cell Brillouin zone challenging. To circumvent this issue, we used supercells with the same number of atoms as those in wrinkled BLG for the phonon dispersion calculations of flat BLG.

Figure 4c,d compares the calculated phonon dispersion for the flat and wrinkled (λ = 1.9 nm) BLG. Apparently, the wrinkling brings about multiple pronounced avoided crossings in the heat‐carrying acoustic branches, with the degree of separation between these bands increasing as the wavelength decreases (dashed circles in Figure 4c, Figure S13, Supporting Information). Additionally, the originally degenerate phonon branches become significantly separated after wrinkling, as highlighted by the dashed rectangles in Figure 4c.

The phonon band avoided crossing and degenerate opening can be attributed to the redistribution of phonon mode polarization vectors in wrinkled BLG.^[^ ^44^ ^]^ Figure 4d,e displays the out‐of‐plane flexural (ZA) and longitudinal acoustic (LA) mode polarization vectors for each atom, projected onto the *X–Z* plane for both flat and wrinkled BLG. The polarization vectors of the ZA and LA modes at 1 and 5 THz are shown as representative examples, with corresponding structural schematics included in each panel. In the flat BLG, the normal modes exhibit well‐defined polarization vectors, with nearly all atoms having a polarization component close to unity in the *Z* (for the ZA mode) or *X* (for the LA mode) direction. In contrast, in the wrinkled BLG, the atomic polarization vectors are distributed along an arc in the *X–Z* plane, as shown in Figure 4e, indicating a significant *X* (for ZA) or *Z* (for LA) component. This suggests that the geometrical effects of wrinkling induce a hybridization of the flexural and longitudinal modes, thereby causing the atomic vibrations to exhibit scattered polarization orientations (Figure S14, Supporting Information). As a result, when the heat flow is perpendicular to the wrinkling direction, the propagating vibrational modes become somewhat localized, which is evidenced by the significant overall downshift in the participation ratio (Figure S15, Supporting Information).

The loss of a preferred polarization direction significantly decelerates phonon bands,^[^ ^45^ ^]^ which in turn impedes phonon transport perpendicular to the wrinkling direction. As evidenced in Figure 4f, the wrinkled graphene exhibits a lower group velocity across the entire frequency range in the direction perpendicular to the wrinkles, compared to its flat counterpart. Notably, the shorter the wrinkling wavelength, the more pronounced the reduction in group velocity. In contrast, the phonon group velocity remains relatively unaffected parallel to the wrinkling direction (Figure 4g). According to the kinetic phonon gas model, the thermal conductivity along a transport direction is calculated as $\kappa =\sum C_{v}\upsilon_{g}^{2}\tau$, where C_v_ is the heat capacity, $\upsilon_{g}$ is the group velocity, and τ is the lifetime. To isolate the effect of group velocity on *κ*_⊥_, we calculated *κ*_⊥_ by varying only $\upsilon_{g}$ while assuming τ remains constant. This calculation reveals that the reduction in $\upsilon_{g}$ alone leads to a significant suppression in *κ*_⊥_, with reductions of 31%, 48%, and 71% for the three wrinkled BLG samples, respectively. Collectively, these results demonstrate that the substantial reduction in *κ*_⊥_ in wrinkled BLG is primarily due to the decreased phonon group velocity caused by geometry‐induced phonon hybridization. To verify the impact of wrinkling on thermal transport, we further performed nonequilibrium MD (NEMD) simulations to calculate the room‐temperature *κ* (Figure 4h). The results show that wrinkling considerably suppresses *κ*_⊥_ whereas slightly affects *κ*_∥_, qualitatively consistent with the variation trend of the results from lattice dynamics calculations. This also accords well with the experimentally measured *κ* under various wrinkling conditions (Figures 2 and 3). It should be noted that the MD domain (<100 nm) is much smaller than the experimental wrinkle dimensions. While this finite‐size effect suppresses the absolute thermal conductivity, all simulations were performed under identical domain sizes, allowing meaningful self‐consistent comparison among different wrinkle configurations. Therefore, the observed relative modulation of heat conduction arises primarily from the effects of wrinkles rather than from finite‐size artifacts.

**Figure 5:**
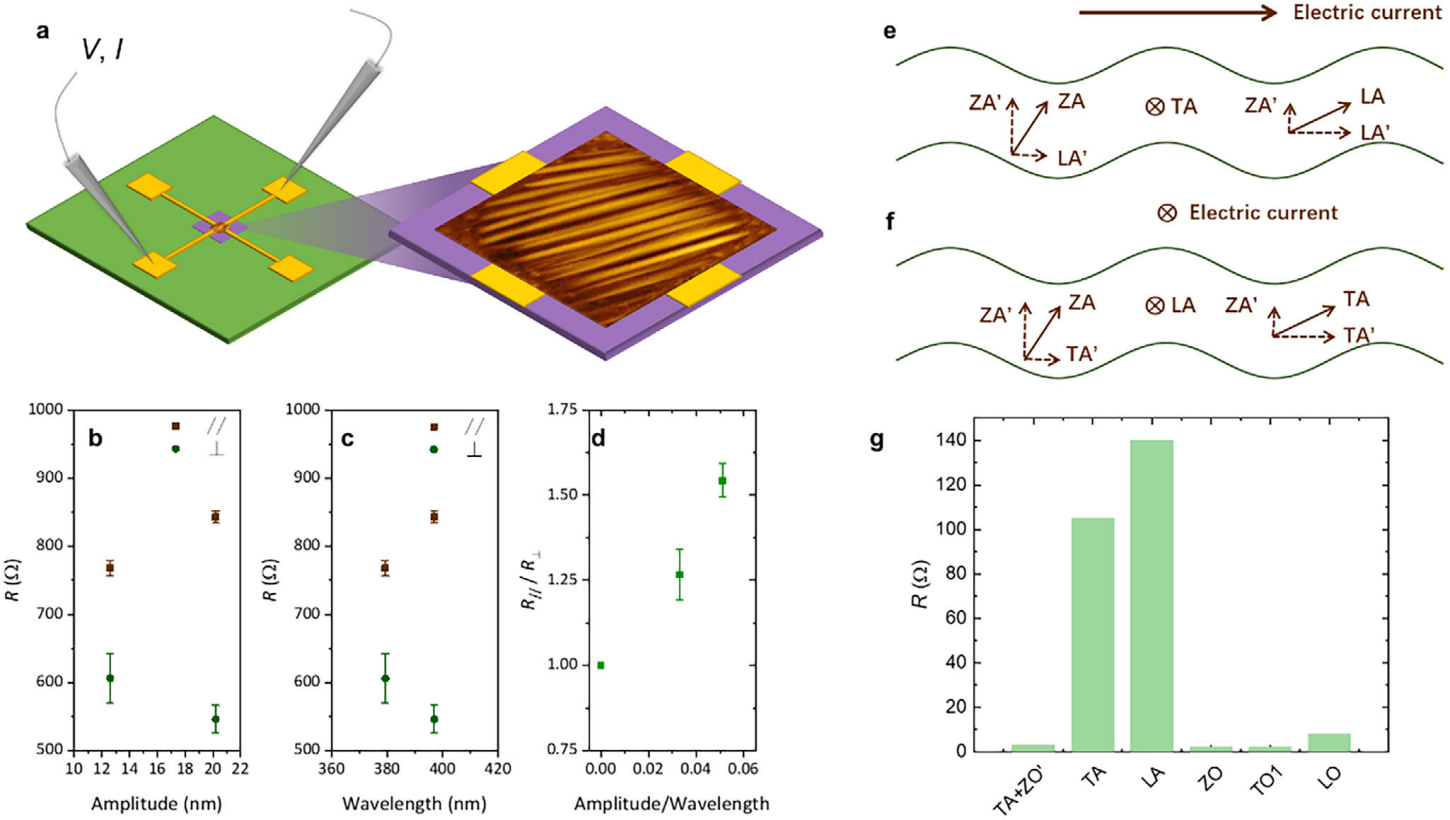
Phonon hybridization induced electrical anisotropy. a) Schematic illustration of the electrical resistance measurement setup for wrinkled BLG. Electrical resistance of wrinkled BLG as a function of b) wrinkling amplitude and c) wrinkling wavelength. d) Anisotropic electrical resistance plotted against the ratio of amplitude over wavelength. Schematics of phonon hybridization when current flows e) perpendicular and f) parallel to the wrinkle direction, showing that ZA, LA, and TA phonons can be decomposed into in‐plane and out‐of‐plane components, facilitating mode hybridization. g) Phonon branch‐resolved contributions to the electrical resistance of pristine BLG at 300 K.

The phonon mode hybridization induced by wrinkling could also introduce anisotropic effects on electrical transport. To investigate this, we measured the electrical resistance of wrinkled BLG using 2‐probe method. As illustrated in **Figure** **5**a, by selecting different pairs of gold electrodes and recording current–voltage (*I–V*) curves, we characterized the resistance along directions parallel and perpendicular to the wrinkles (see Experimental Section, Supplementary Note 5, Supporting Information). Figure 5b,c presents the measured electrical resistance as functions of wrinkling amplitude and wavelength, respectively, for two BLG samples with distinct wrinkle configurations (Figure S16, Supporting Information). Compared to flat graphene,^[^ ^46^ ^]^ the wrinkled samples exhibited a noticeable increase in resistance, consistent with previous reports.^[^ ^7^ ^]^ Notably, as shown in Figure 5d, the resistance displays pronounced anisotropy, with the parallel resistance exceeding that of the perpendicular direction. This behavior contrasts with the thermal anisotropy observed earlier, where thermal transport is more resistive perpendicular to the wrinkles than along them, highlighting the decoupling of electrical and thermal transport in wrinkled BLG.

This unexpected phenomenon may stem from geometric effects of wrinkles that modulate electron‐phonon scattering, thereby governing the phonon‐limited electrical resistance. In pristine flat BLG, room‐temperature resistivity is primarily due to electron scattering by acoustic phonons, with the LA modes exhibiting considerably larger contributions than the TA modes, as quantitatively revealed by the phonon branch‐resolved contribution in Figure 5g. As discussed above, the introduction of wrinkles induces hybridization between out‐of‐plane flexural ZA phonons and in‐plane modes, which affects charge carrier lifetimes through modulating electron‐phonon scattering. While quantitatively calculating resistivity in wrinkled BLG remains challenging due to structural and computational complexities,^[^ ^47^, ^48^ ^]^ we propose an intuitive physical picture to explain the observed electrical anisotropy. As schematically depicted in Figure 5e, the resistivity anisotropy arises from current‐direction‐dependent phonon hybridization. When current flows perpendicular to the wrinkles, ZA phonons hybridize with LA phonons, while TA phonons remain largely unaffected. Conversely, with current parallel to the wrinkles, ZA phonons predominantly hybridize with TA modes, leaving LA modes decoupled. This indicates that LA modes are considerably suppressed by geometric effects when current flows perpendicular to wrinkles, whereas TA modes become more susceptible to such effects under parallel current flow. Given that LA phonons contribute significantly more to resistivity than TA phonons (Figure 5g), the direction‐dependent phonon hybridization results in lower resistance perpendicular to wrinkles compared to the parallel direction.

This study establishes a clear experimental framework and mechanistic understanding of wrinkling‐engineered transport phenomena based on bilayer graphene, opening avenues for directional and decoupled control of heat and charge flow in various 2D materials. Beyond fundamental interest, such control could be exploited in practical applications: for instance, anisotropic thermal management in ultrathin electronics, wrinkling‐based tuning of carrier scattering for thermoelectric optimization, or decoupled heat/charge pathways in 2D material‐based logic circuits and interconnects.

## 3 Conclusion

In summary, we developed a novel sample preparation method that effectively eliminates lattice strain, enabling the isolation of pure geometric distortion effects on thermal and electrical transport in wrinkled bilayer graphene. Our measurements reveal a strikingly inverse anisotropic relationship: *κ*_⊥_ is lower than *κ*_∥_, whereas electrical resistance exhibits the opposite trend, with *R*_⊥_ lower than *R*_∥_. This clearly demonstrates the decoupling of thermal and electrical transport induced by wrinkling. The suppression of *κ*_⊥_ was further confirmed by applying pressure via bulging, which flattened the wrinkles and restored the intrinsic thermal conductivity. Atomistic simulations attribute these effects to phonon mode hybridization caused by out‐of‐plane geometric perturbations. For thermal transport, phonon hybridization induces band avoided crossings and degeneracy lifting, erasing preferred phonon polarization directions and reducing phonon group velocities perpendicular to the wrinkles. For electrical transport, geometric perturbations promote distinct hybridization patterns between out‐of‐plane flexural (ZA) phonons and in‐plane (LA and TA) modes, with the differing contributions of LA and TA phonons to resistivity leading to anisotropic electrical transport. This work opens new avenues for exploring wrinkling‐engineered transport phenomena, and offers a promising strategy for directionally and independently controlling heat and charge flow in the broader class of 2D materials.

## 4 Experimental Section

### Sample Preparation

HOPG was purchased from HQ Graphene. Bilayer graphene was first micro‐mechanically exfoliated onto SiO_2_/Si substrate, and then transferred onto the PDMS stamp using water‐assisted transfer method. Finally, the bilayer graphene on the PDMS is stretched and released onto the patterned SiO_2_/Si substrate to form suspended wrinkles. Patterned SiO_2_/Si substrate with 3 and 5.4 µm diameters array of circle holes was fabricated by photolithography and reactive ion beam etching. The depth of the cylindrical microcavities is 300 nm.

### AFM Measurement

The MFP‐3D AFM was used to characterize the morphology of graphene by using AFM tips (OLTESPA OMCL‐AC240TS) with tetrahedral geometry, and the nominal tip radius was ≈7 nm.

### Thermal Conductivity Measurements Using Micro Raman

The optical path of the probing laser passes through a scanning galvanometer, and by changing the angle of the scanning galvanometer, the relative position of the probing laser and the heating laser can be changed, thus the temperature distribution caused by the heating laser can be determined by the Raman peak shift at different positions. The wavelength of the heating laser was 532 nm, and the probing laser was 633 nm. The numerical aperture of the objective lens was 0.8. The typical Raman spectra of flat and wrinkled sample can be found in Figure S4 (Supporting Information). Due to wrinkles affecting in‐plane vibration, the 2D peak of wrinkled sample at ≈2700 cm^−1^ was weakened and widened, with an obvious displacement compared with that of the flat sample. In contrast, the G peaks at ≈1680 cm^−1^, reflecting the C─C bond vibration, remain nearly unchanged. Therefore, the Raman shift of G peak was used to determine the temperature variation, and the coefficient was determined to be −0.024 cm^−1^ K^−1^ (Figure S5, Supporting Information).

### Electrical Resistance Measurements

The electrical resistance of wrinkled BLG using 2‐probe method was measured. As shown in Figure 5a, the sample was suspended on a square notch etched into a SiO_2_/Si surface. Four gold pads were connected to the four sides of the wrinkled BLG sample, serving as the electrodes. For wrinkled graphene sample, we choose different combinations of gold pads to measure the current–voltage (*I–V*) curves, the resistance parallel and perpendicular to the wrinkle can be characterized by the slopes of the *I–V* curves. Two samples with different wrinkling configurations were measured and the details can be found in Figure S16 (Supporting Information).

### Atomic Structure Modeling Of Wrinkled Graphene

The atomic structures of wrinkled graphene were generated by performing non‐equilibrium molecular dynamics (NEMD) simulations using the LAMMPS package,^[^ ^49^ ^]^ with a velocity Verlet algorithm for numerical integration of the equations of motion and a time step of 0.5 fs. The initial atomic structure of bilayer graphene sheet was built with 10 × 2 × 1 conventional cells containing 160 atoms. A vacuum spacing with the thickness of 20 Å was adopted to avoid the effect of mirror interactions between the layers. The in‐plane interatomic interactions between carbon atoms were described by the AIREBO potential,^[^ ^50^ ^]^ and the Van der Waals interactions between layers were characterized by the Kolmogorov‐Crespi potential.^[^ ^51^, ^52^ ^]^ The initial system with periodic boundary conditions (PBC) was relaxed to reach thermal equilibrium at 300 K and zero pressure in the isothermal‐isobaric (NPT) ensemble using the Nosé‐Hoover thermostat. Then the system was progressively subjected to an in‐plane compressive strain along the long (*x*) axis, during which the wrinkles in the experimentally observed samples was reproduced perpendicular to the *x* axis, and the resultant wrinkle wavelength and out‐of‐plane amplitude is dependent on the compressive strain. Using this structure as a unit cell, the periodic wrinkles in suspended graphene can be formed by expanding the cell along the in‐plane directions.

### Phonon Dispersion Calculations

Phonon dispersion curves throughout the Brillouin zone were obtained by diagonalizing the dynamical matrix via the Phonopy package,^[^ ^53^ ^]^ which was computed by the Fourier transform of second‐order interatomic force constants (IFCs). The unit cell of pristine bilayer graphene involves four atoms, but the presence of wrinkles breaks its lattice periodicity, resulting in the periodic wrinkle unit cell containing many atoms. Hence, exact calculating the 2nd‐IFCs of wrinkled graphene from density functional theory becomes unfeasible. In light of this, here we calculated the 2nd‐IFCs with 2 × 2 × 1 supercells containing 640 atoms via the finite displacement method, using the classical potential mentioned above to describe the interatomic interactions. To characterize the degree of localization for each mode, the participation ratio (PR) was also calculated, ^[^ ^54^, ^55^ ^]^ which is defined as $\mathrm{PR}=\frac{{\left(\sum_{i=1}^{N}\left(\sum_{\alpha =1}^{3}u_{i\alpha }^{2}\right)\right)}^{2}}{\sum_{i=1}^{N}{\left(\sum_{\alpha =1}^{3}u_{i\alpha }^{2}\right)}^{2}}$, where *N* is the number of atoms and u_iα_ is the eigenvector component for atom *i* in the direction *α*. For a fully delocalized mode, PR = 1, while for a mode that is fully localized on a single atomic site, PR approaches zero.

### Thermal Conductivity Calculations

In this work, the NEMD simulations were conducted to calculate the lattice thermal conductivity (*κ*), with the domain size of 96 × 84 × 6 nm^3^ containing 80 000 atoms. First, we performed simulations at 300 K in the canonical (NVT) ensemble for 50 ps to help the system reach thermal equilibrium. Then the simulations were switched to the canonical (NVE) ensemble to calculate the room‐temperature *κ* of wrinkled graphene models for a further 200 ps, which is long enough to bring the systems to the stationary state and obtain the corresponding heat flux (*J*) and temperature gradient along the *x* direction (*∇T*). The temperatures at the source and sink regions of the simulation domains were controlled with Langevin thermostats.^[^ ^56^, ^57^ ^]^ According to Fourier's law, the lattice thermal conductivity is determined by κ = −*J*/∇T.

### Electrical Resistivity Calculations

To determine the electrical resistivity of pristine flat BLG, the electron energies and phonon frequencies were calculated using the Quantum‐ESPRESSO package^[^ ^58^ ^]^ based on density‐functional theory (DFT) and density‐functional perturbation theory (DFPT). Norm‐conserving pseudo‐potentials^[^ ^59^ ^]^ and a plane‐wave basis set with the kinetic energy cutoff of 60 Ry^[^ ^60^ ^]^ were employed. The electron‐phonon coupling matrix elements were then calculated using the EPW package,^[^ ^61^ ^]^ with the coarse grids of 8 × 8 × 1 for both k and q points. These were subsequently interpolated onto a dense grid of 500 × 500 × 1 to compute the electron‐phonon scattering rates and electrical resistivity using our modified EPW package.^[^ ^47^, ^48^ ^]^

## Conflict of Interest

The authors declare no conflict of interest.

## Author Contributions

A.F. and W.D. contributed equally to this work. L.Y. proposed and directed the research; W.D. prepared the wrinkled bilayer graphene samples, performed the bulging tests and the strain characterizations using Raman; A.F. conducted the thermal conductivity measurements and temperature/strain mapping of the bilayer graphene samples using Raman spectroscopy; X.Y. and Y.H. performed the theoretical modeling; the manuscript was prepared by L.Y., W.D., A.F., X.Y., L.L., and X.Z. with input from all co‐authors.

## Supporting information



Supporting Information

## Data Availability

The data that support the findings of this study are available in the supplementary material of this article.
