# Supplementary material for: Decoupling Heat and Electrical Conduction in Bilayer Graphene Through Wrinkling‐Induced Phonon Hybridization
Source: Adv Sci (Weinh). 2025 Oct 30;13(3):e16792. doi: 10.1002/advs.202516792 (PMC12806555; doi:10.1002/advs.202516792)
Supplement: Supplementary file 1 — Supporting Information [file ADVS-13-e16792-s001.docx]

**Supplementary Information**

for

**Decoupling heat and electrical conduction in bilayer graphene through wrinkling-induced phonon hybridization**

Aoran Fan^1,*^, Wenlong Dong^2,*^, Xiaolong Yang^3,†^, Ya Hu^3^, Yufeng Zhang^1^, Wu Li^4^, Jun Lyu^5^, Luqi Liu^2,†^, Xing Zhang^1,†^, Lin Yang^5,6,†^

^1^Department of Engineering Mechanics, Tsinghua University, Beijing 100084, China

^2^CAS Key Laboratory of Nanosystem and Hierarchical Fabrication, National Center for Nanoscience and Technology, Beijing 100190, China

^3^College of Physics, and Center of Quantum Materials and Devices, Chongqing University, Chongqing 401331, China

^4^Eastern Institute for Advanced Study, Eastern Institute of Technology, Ningbo 315200, China

^5^Department of Advanced Manufacturing and Robotics, College of Engineering, Peking University, Beijing 100871, China

^6^National Key Laboratory of Advanced Micro and Nano Manufacture Technology, Peking University, Beijing 100871, China

*: These authors contributed equally to this work.

†: Authors to whom correspondence should be addressed.

Email: [yangxl@cqu.edu.cn](mailto:yangxl@cqu.edu.cn), [liulq@nanoctr.cn](mailto:liulq@nanoctr.cn), [x-zhang@tsinghua.edu.cn](mailto:x-zhang@tsinghua.edu.cn), [linyangpku@pku.edu.cn](mailto:linyangpku@pku.edu.cn)

**Supplementary Note 1. Strain calculations for bulged BLG**

We subjected suspended graphene to biaxial strain loading using a bulging device. Such bulging devices, commonly known as bubble testing, have been widely employed to investigate the mechanical, interfacial, and strain-dependent properties of 2D thin films. By introducing gas diffusion to create a pressure difference (Δ*p*) between the sealed cavity shown in Fig. S1 and the external environment, suspended graphene is pushed upward, thereby undergoing continuous strain loading via a controlled expansion process. Under Δ*p*, the suspended BLG expands, and the central cross-sectional profile satisfies thin film assumptions, allowing to neglect the contribution of bending stiffness:

$z=h(1-\frac{x^{2}}{a^{2}})$

where $z$ represents the out-of-plane deflection, $x$ represents radial positions of the bubble, $h$ is the central height of the bubble, and $a$ denotes the bubble radius. Consequently, the loading strain at the bubble center point can be determined based on the extracted $h$ and $a$ values from the central cross-sectional profile:

$\varepsilon_{x}=\varepsilon_{\theta}=\left\{ \begin{aligned} &\frac{3-\nu}{4}\frac{h^{2}}{a^{2}},strong-shearlimit \\ &\frac{1-\nu}{2}\frac{h^{2}}{a^{2}},weak-shearlimit \end{aligned} \right.$

It's important to note that the strain of 2D material bubbles is influenced by both mechanical properties (e.g., Young's modulus and Poisson's ratio) and the interfacial conditions (e.g., interfacial shear strength). Given the weak shear interaction (interlayer shear stress ~ 40 kPa) between graphene layers, the strain of BLG tends towards the weak shear limit.


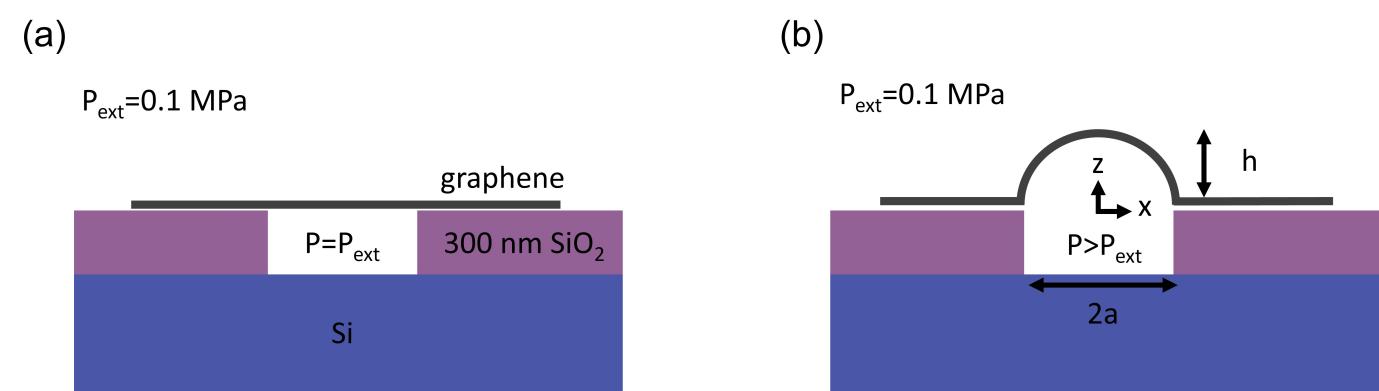


**Figure S1.** Schematic cross-sectional diagram of the bulging device at $\Delta p=0$ (a) and $\Delta p>0$ (b), respectively.

**Supplementary Note 2. Strain characterization in wrinkled BLG**

We loaded flat BLG with bulging strain and measured Raman spectra at the corresponding strains. Through the strain calculated by Equation (2) and the recorded Raman peak positions of G band, we can get Gruneisen parameter (γ) of BLG. For biaxial strain, γ can be written as:

$\gamma=\frac{1}{2\omega_{G}^{0}}\frac{d\omega_{G}}{d\varepsilon}$

Where $\varepsilon$ is the loading strain, $\omega_{G}$ is the peak position of G band under strain, and $\omega_{G}^{0}$ is the initial peak position of BLG. In turn, with known peak shifts of wrinkled BLG and γ of BLG, we can determine the initial pre-strain as well as the strain by bulging at $\Delta p$ of the wrinkled BLG.

**
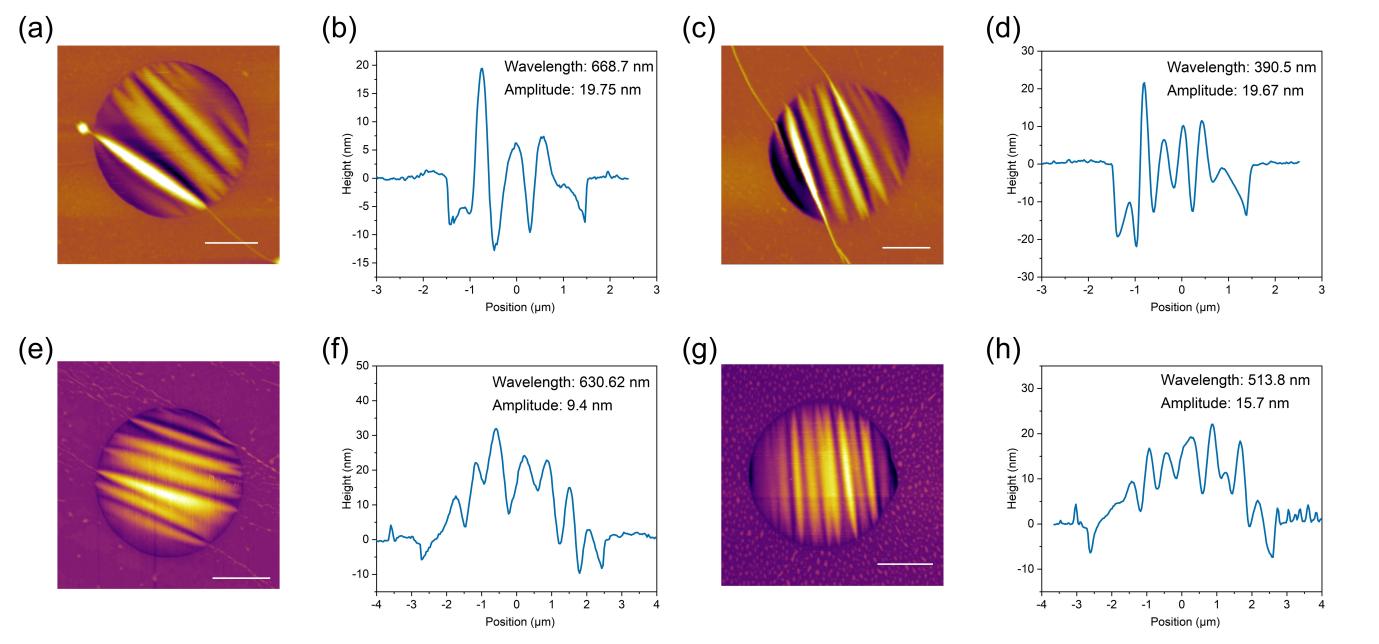
**

**Figure S2.** Morphology of wrinkled BLG. AFM images and cross-section profiles of four wrinkled BLG with varied amplitude and wavelength. Scale bar: 1 μm (a, c) and 2 μm (e, g).


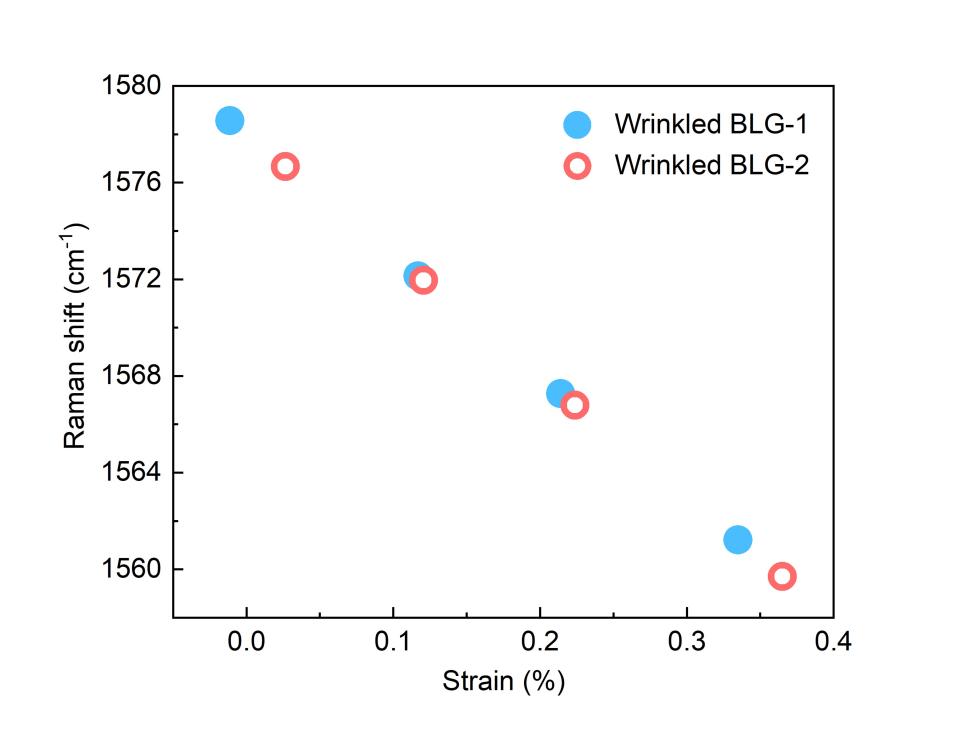


**Figure S3.** The Raman G band shifts of wrinkled BLG in response of loading strain.

**Supplementary Note 3.** **Thermal conductivity measurement method**

As shown in Fig. 2a in the main manuscript, a Gaussian laser beam is used to heat the sample, and another Gaussian laser beam is used as the probe of Raman spectroscopy measurement. Due to the different wavelength of the heating laser and the probing laser, the Raman spectra excited by the heating pulse and the probing pulse can be distinguished. The Raman scattering excited by heating pulse can be eliminated through changing the grating position of Raman spectrometer or using an appropriate cut-off filter in the measurement. Thus, the temperature distribution of the sample can be determined by the Raman peak. The power of the probing laser was much weaker than it of the heating laser. Furthermore, due to the identical influence of the probing pulse of each measuring data, the weak heating effect of the probing laser can be eliminated by analyzing the temperature variations.

$\kappa_{x}\frac{d^{2}T}{dx^{2}}+\kappa_{y}\frac{d^{2}T}{dy^{2}}+q\left( x,y \right)=0$ (4)

where,

$q\left( x,y \right)=\frac{\eta q_{0}}{\delta}\exp\left( -\frac{\left( x-x_{0} \right)^{2}+\left( y-y_{0} \right)^{2}}{r_{0}^{2}} \right)$ (5)

The heat conduction process of the suspended 2D nanomaterial sample under heating can be expressed as Eq. (4), where *T* is the temperature rise of the sample at position (*x*, *y*), *κ_x_* and *κ_y_* are the thermal conductivities in different direction of the sample, *η* is the laser absorptivity, *δ* is the thickness of the 2D nanomaterial sample, *q*_0_ is the laser power density of the heating pulse at the beam center (*x*_0_, *y*_0_), *r*_0_ is the laser spot radius of the heating laser where the power density attenuates to *q*_0_/e.

Normalized with a characteristic temperature $\theta_{0}=\eta q_{0}l^{2}/\delta\kappa_{x}$, the Eq. (4) can be rewritten as:

$\frac{d^{2}\theta}{dx^{2}}+\frac{\kappa_{y}}{\kappa_{x}}\frac{d^{2}\theta}{dy^{2}}+\exp\left( -\frac{\left( x-x_{0} \right)^{2}+\left( y-y_{0} \right)^{2}}{r_{0}^{2}} \right)=0$ (6)

where *x=x/l* and *y=y/l* is the dimensionless coordinate with the characteristic length, *l*, *θ= T/θ_0_* is the dimensionless temperature rises of the sample. And it is easy to find that the dimensionless temperature rises are only determined by the ratio of the *κ_y_* and *κ_x_*. And the certain value of the thermal conductivity can be further determined by the real temperature rises.

For the flat BLG sample, when the *κ_y_* is equal to *κ_x_*, the real temperature rises are only determined by the thermal conductivity and the heating laser. Therefore, the thermal conductivity of flat BLG can be calculated by the temperature rise versus heating laser power.


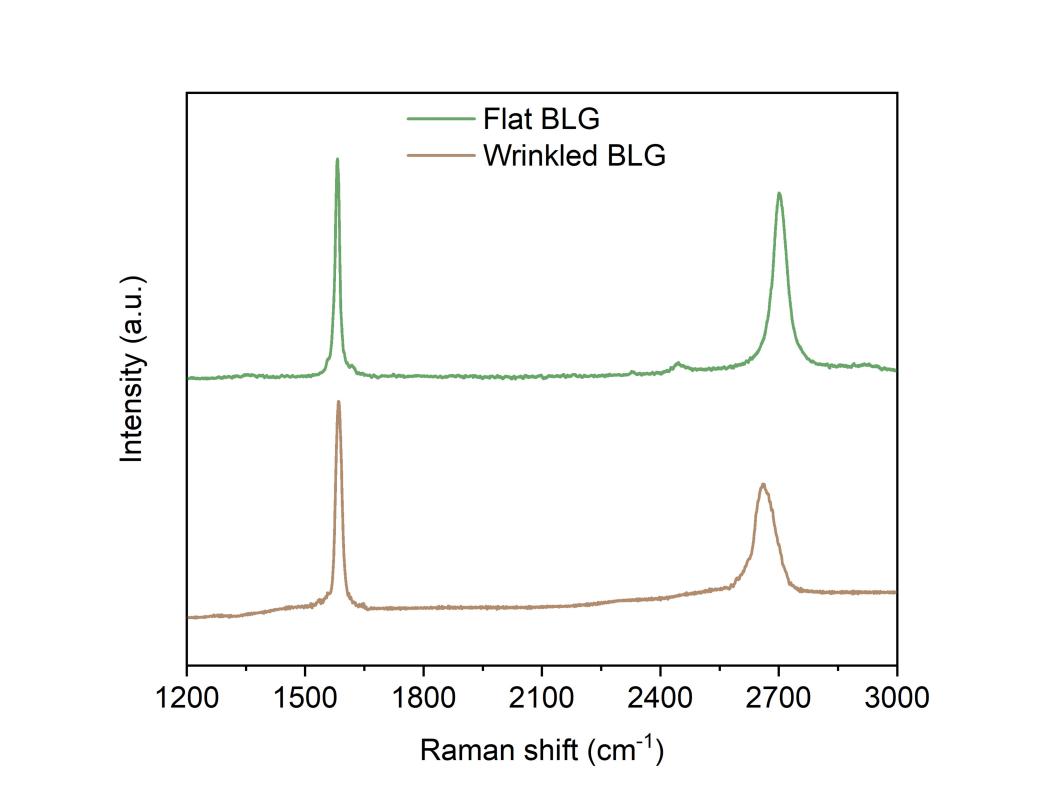


**Figure S4.** Raman spectra of flat BLG and wrinkled BLG.


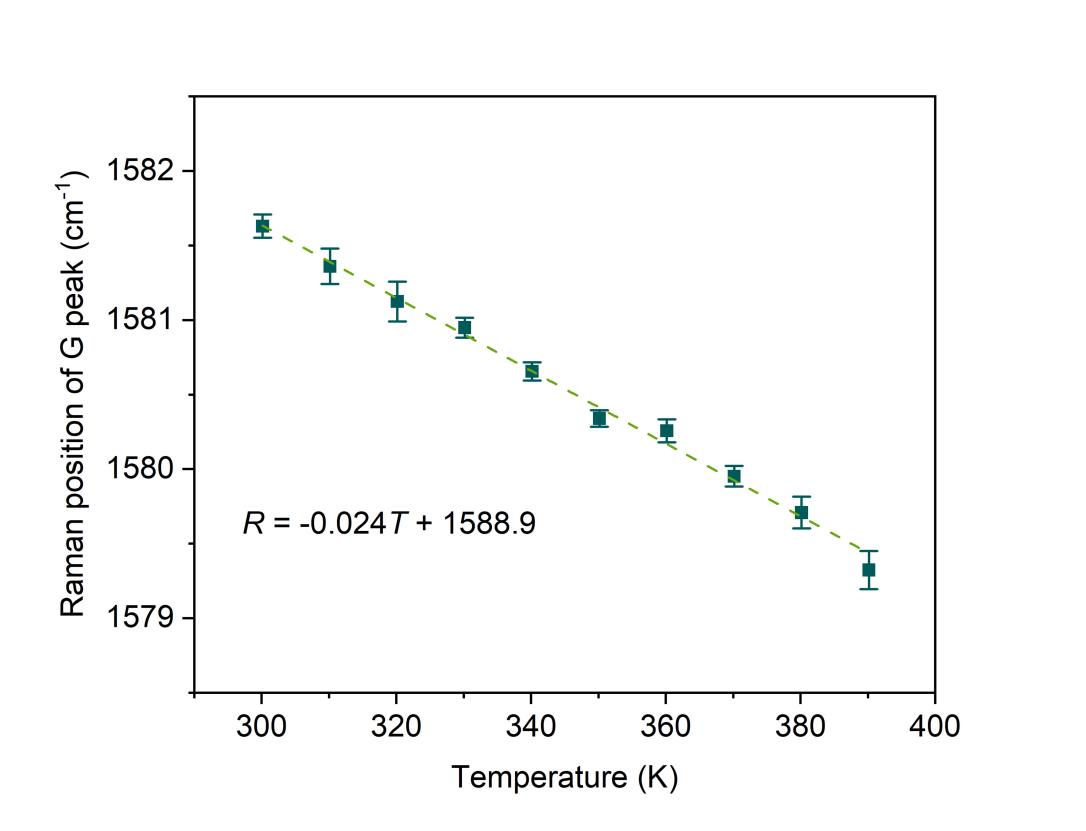


**Figure S5.** Raman peak shift versus temperature rise.


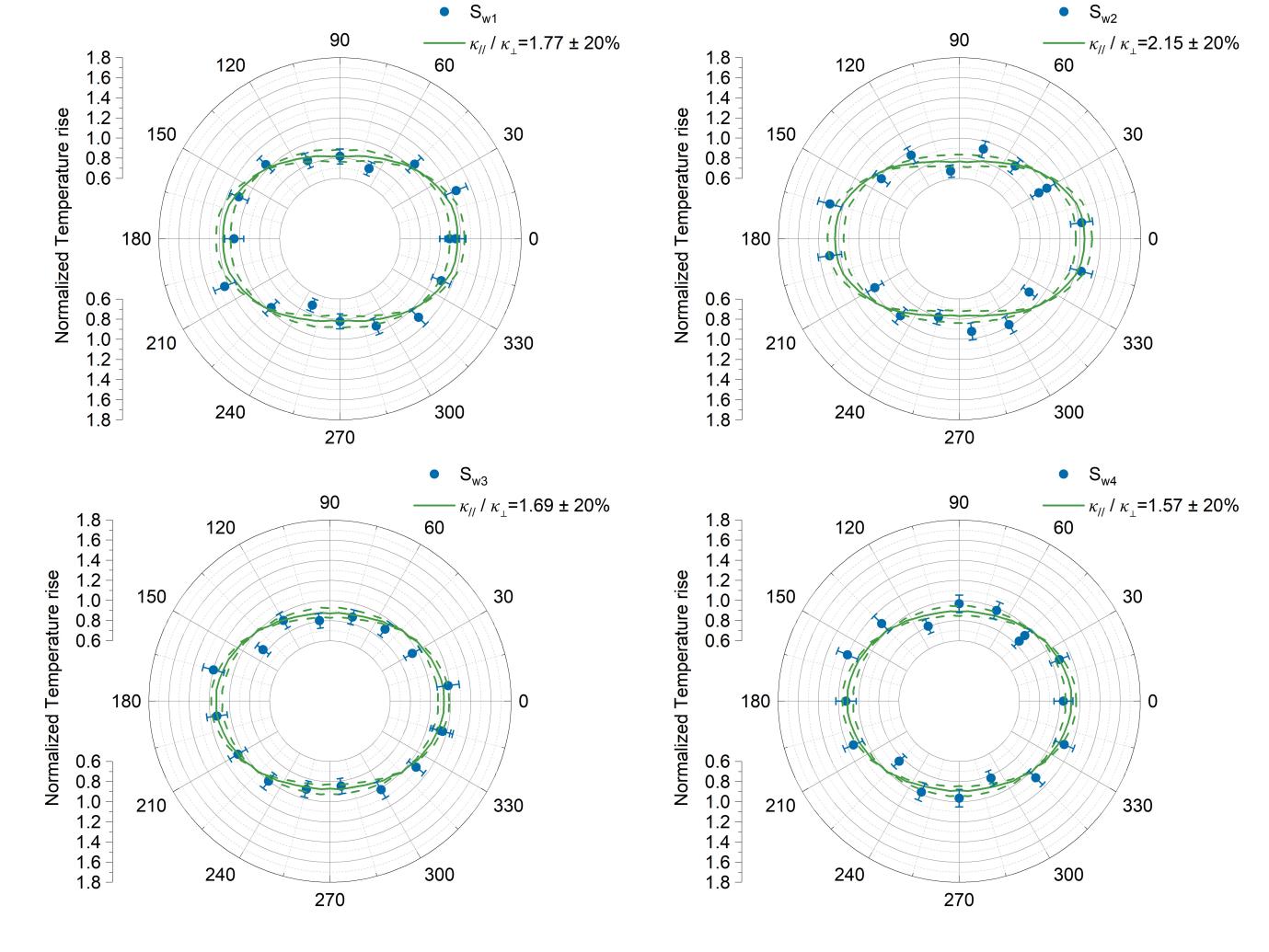


**Figure S6.** Temperature variation of wrinkled BLG.


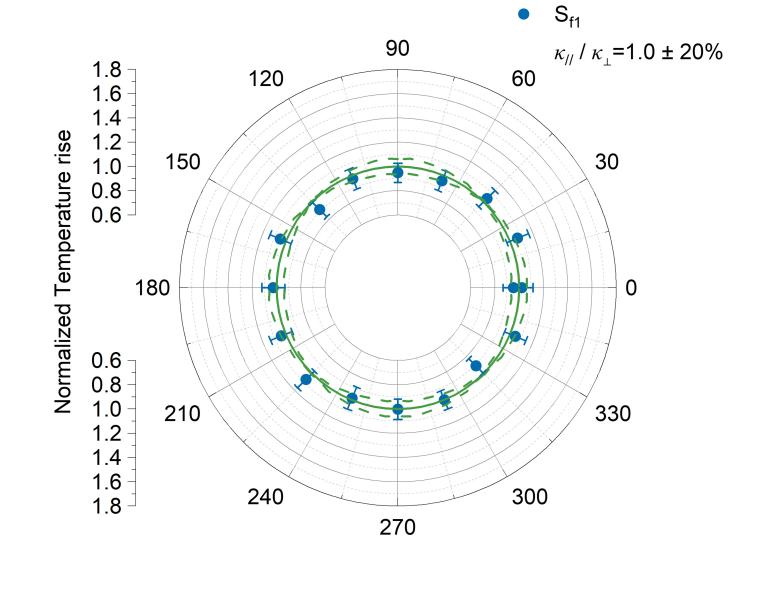


**Figure S7.** Temperature variation of flat BLG.

**Table S1** Measurement information of wrinkled BLG

| Sample | *D_sample_* / μm | *D_heating-laser_* / nm | *D_probing-laser_* / nm | Wavelength / nm | Amplitude / nm |
| --- | --- | --- | --- | --- | --- |
| S_w1_ | 3.0 | 423.3 | 503.7 | 668.70 | 19.75 |
| S_w2_ | 3.0 | 423.3 | 503.7 | 390.50 | 19.67 |
| S_w3_ | 5.4 | 423.3 | 503.7 | 630.62 | 9.40 |
| S_w4_ | 5.4 | 423.3 | 503.7 | 513.80 | 15.70 |

**Table S2** Thermal conductivity measurement results of wrinkled BLG

| Sample | *Q* / mW | *R*_Measurement_ / μm | *κ*_//_ / *κ*_⊥_ | *T_ave_* / K | *κ*_⊥_ / Wm^-1^K^-1^ | *κ_e_* / Wm^-1^K^-1^ |
| --- | --- | --- | --- | --- | --- | --- |
| S_w1_ | 10.7 | 1.0 | 1.77 ±0.12 | 45.9±0.4 | 613±48 | 786±62 |
| S_w2_ | 10.7 | 1.0 | 2.15 ±0.15 | 50.4±0.4 | 485±38 | 743±59 |
| S_w3_ | 4.7 | 1.0 | 1.69 ±0.12 | 47.0±0.4 | 633±50 | 835±66 |
| S_w4_ | 4.7 | 1.0 | 1.57 ±0.11 | 40.8±0.3 | 757±60 | 962±76 |

**Table S3** Thermal conductivity measurement results of flat BLG

| Sample | *D* / μm | *κ* / Wm^-1^K^-1^ |
| --- | --- | --- |
| S_f1_ | 3.0 | 1187±94 |
| S_f2_ | 5.4 | 1031±81 |
| S_f3_ | 5.4 | 1051±83 |
| S_f4_ | 5.4 | 1106±87 |

**Table S4** Thermal conductivity measurement results of flat BLG S_f4_ at different strain.

| Sample | Strain / % | *κ* / Wm^-1^K^-1^ |
| --- | --- | --- |
| S_f4_ | 0.00 | 1106±87 |
| S_f4_ | 0.10 | 1186±94 |
| S_f4_ | 0.23 | 1199±95 |
| S_f4_ | 0.37 | 1227±97 |


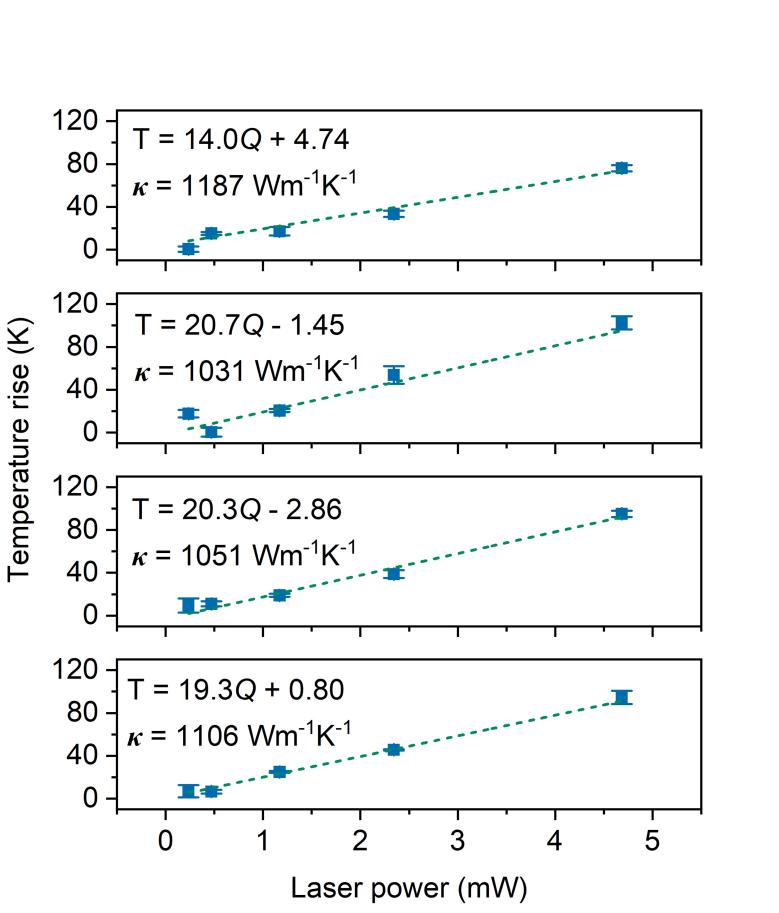


**Figure S8.** Thermal conductivity measurement results of flat BLG.


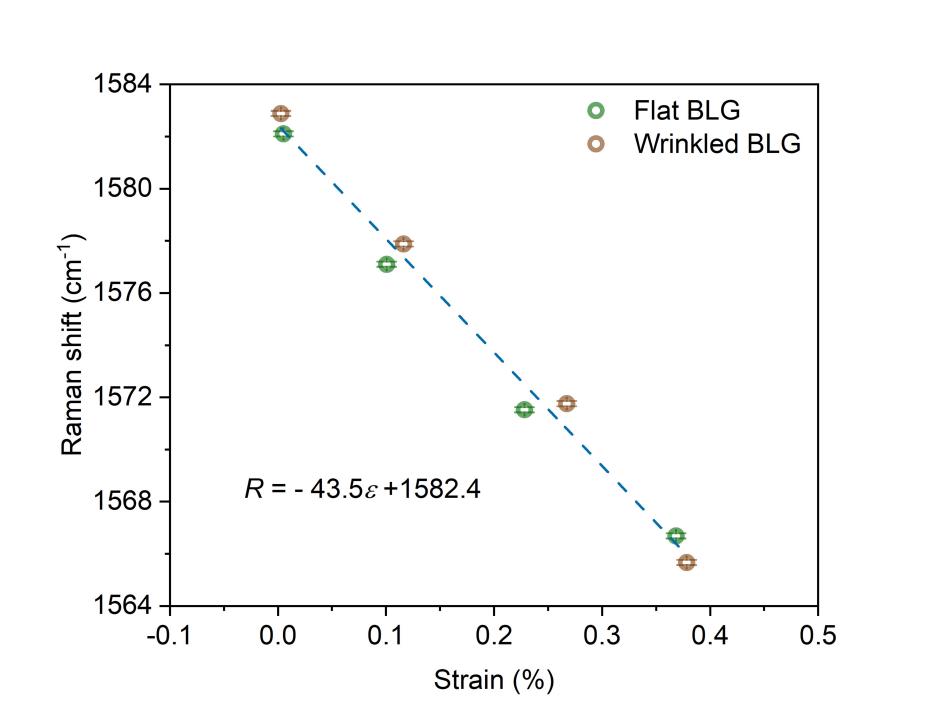


**Figure S9.** Raman shift of G peak versus strain.


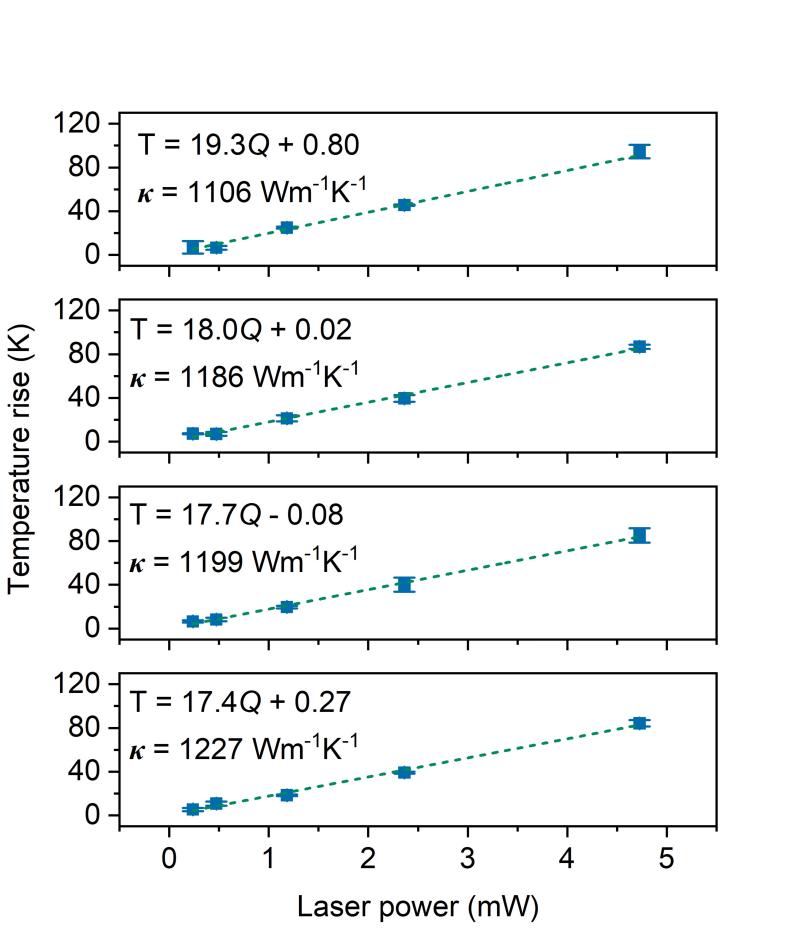


**Figure S10.** Thermal conductivity measurement results of flat BLG S_f4_ at different strain.

**Supplementary Note 4. Thermal Conductivity Uncertainty Analysis**

Measurement uncertainty of thermal conductivity measurement is analyzed with over hundred virtual experiments, and the virtual experimental data *VD*(*β*) is composed of the true temperature rise calculated by true thermal conductivity and the random measurement error, as shown in Eq. (7).

$VD\left( \beta\right)=T\left( \kappa_{x},\kappa_{y},\beta\right)+\frac{\sum_{i=1}^{M} N_{i}\left( 0,\left( \delta T \right)^{2} \right)}{M}$ (7)

Where *T*(*κ_x_*, *κ_y_*, *β*) is the true temperature rise at angle *β* calculated with the true thermal conductivities, *κ_x_* and *κ_y_*. *N_i_*(0,(*δT*)^2^) represents the random temperature measurement error, which is a random number of a normal distribution with the mathematical expectation of 0 and variance of (*δT*)^2^, *δT* is the standard deviation of temperature measurement, which represents the uncertainty of the temperature measurement, and *M* is the times of virtual repeat measurement. In this case, thermal conductivities, *κ_x_*, and *κ_y_*, can be determined by 2 steps: firstly, obtain the ratio of the thermal conductivities, *κ_x_*/*κ_y_*, by the best fit curves of the normalized temperature distribution with a normalization characteristic of average temperature rise; secondly, determine the *κ_x_* and *κ_y_* with the real average temperature rise. Therefore, *κ_x_*/*κ_y_* can be determined by the least square method with Eq. (8).

$\sum_{i=1}^{N^{e}} \left( \frac{VD\left( \beta\right)}{VD_{ave}}-\frac{T\left( \beta\right)}{T_{ave}} \right)\frac{\partial T\left( \kappa_{x}/\kappa_{y} \right)}{T_{ave}\partial\left( \kappa_{y}/\kappa_{y} \right)}=0$ (8)

where *N^e^* is the number of virtual measurements. The measurement error of the virtual experiment is the relative difference between the measured (*κ_x_/κ_y_*)^m^ and the true (*κ_x_/κ_y_*)^*^, which is equal to [(*κ_x_/κ_y_*)^*^_-_ (*κ_x_/κ_y_*)^m^]/(*κ_x_/κ_y_*)^*^_._ By a sufficient number of the virtual experiments, most of the real experimental results will be covered in the virtual analysis. Thus, the maximum measurement error of the sufficient virtual experiments can be perceived as the uncertainty of the measurement.

Assuming *κ_x_/κ_y_* = 1.50, one set of virtual data can be plot in Figure S11a, with a temperature measurement uncertainty of ±3 K and an average temperature rise of 50 K, which is determined by our experimental data, and the number of virtual measurement points *N^e^* is 16, times of repeat measurement *M* of each point is 3, the measurement error of *κ_x_/κ_y_* of 1000 virtual experiments can be shown in Figure S11b. It can be found, 99.9% of the measurement error within ±7%, therefore, the 3σ uncertainty of *κ_x_/κ_y_* is ±7%. The measurement uncertainty of average temperature rise can be determined by Eq. (9), which is about ±0.87%. Thus, the measurement uncertainty of thermal conductivities is about ±7.9%.

$\delta\left( T_{ave} \right)=\frac{\delta T}{\sqrt{M\times N^{e}}}$ (9)


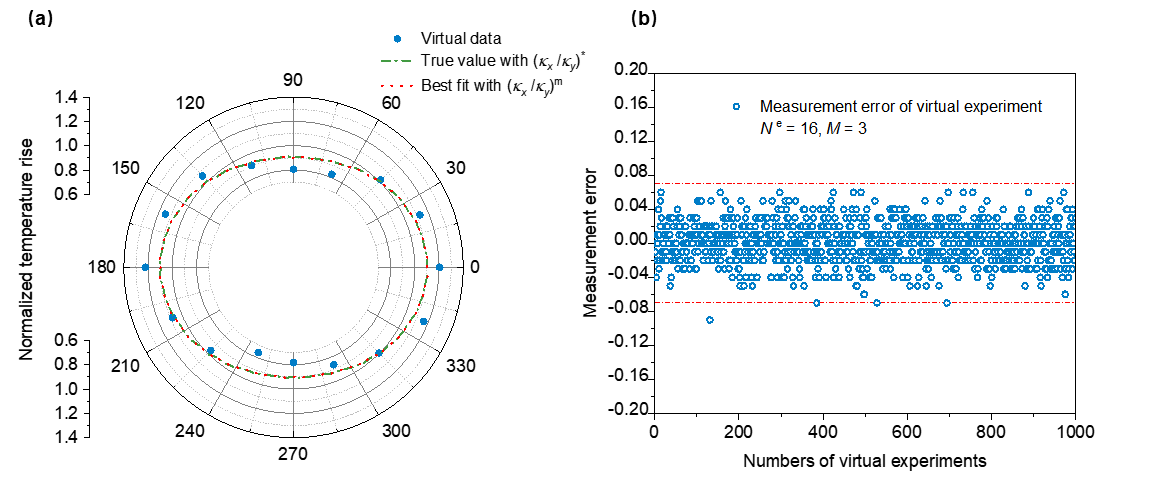


**Figure S11.** (a) Schematic of the uncertainty analysis with the virtual experiment data. (b) Measurement error of *κ_x_/κ_y_* of 1000 virtual experiments.


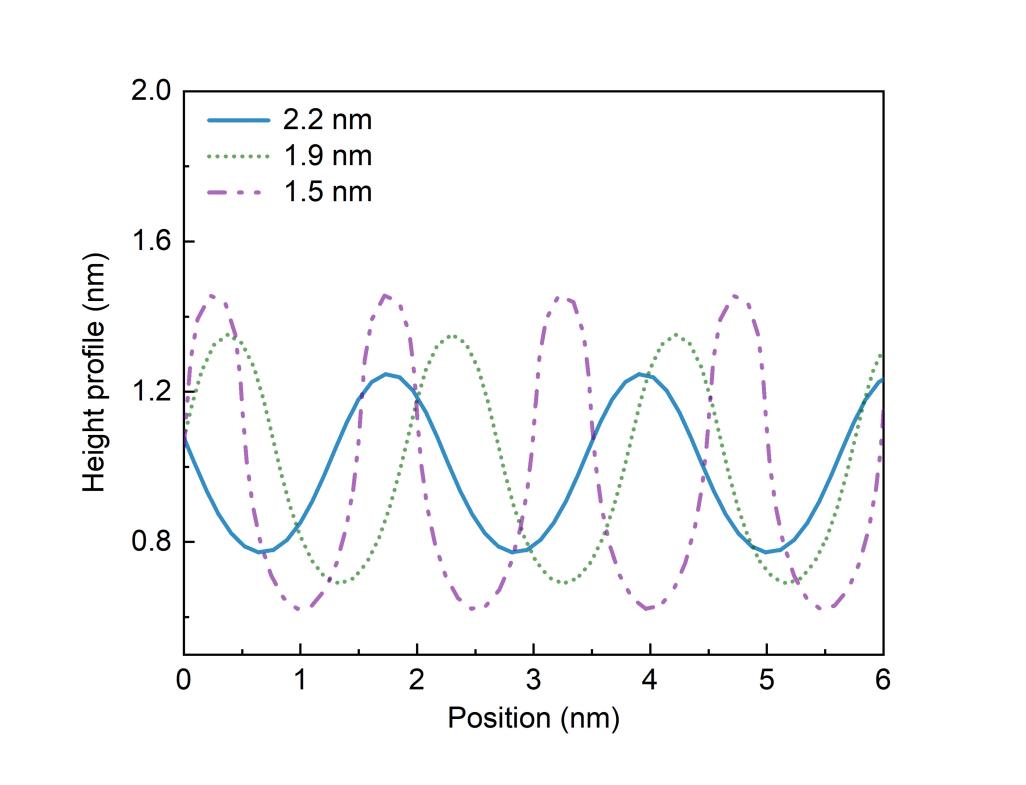


**Figure S12.** Height profiles of wrinkled graphene sheets with different wavelengths.


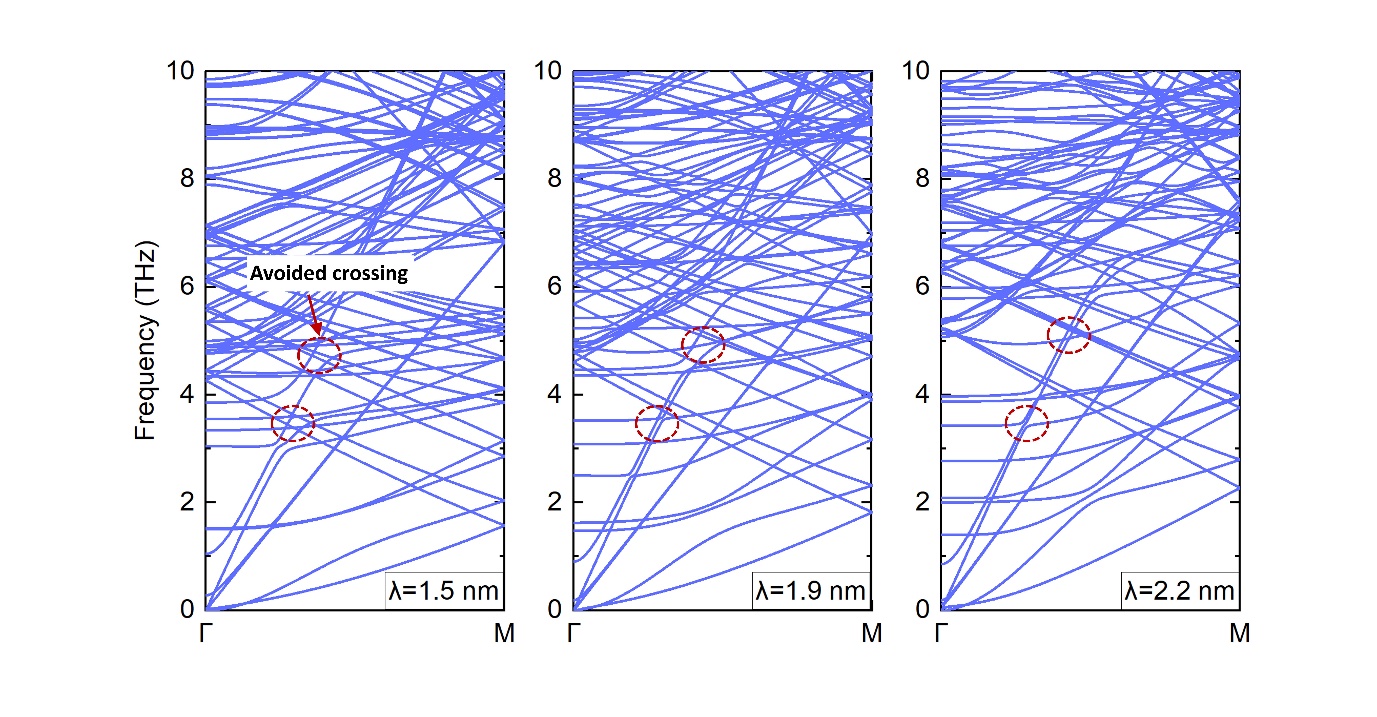
**Figure S13.** Zoom-in plots of the calculated phonon dispersions for wrinkled BLG with different wavelengths.


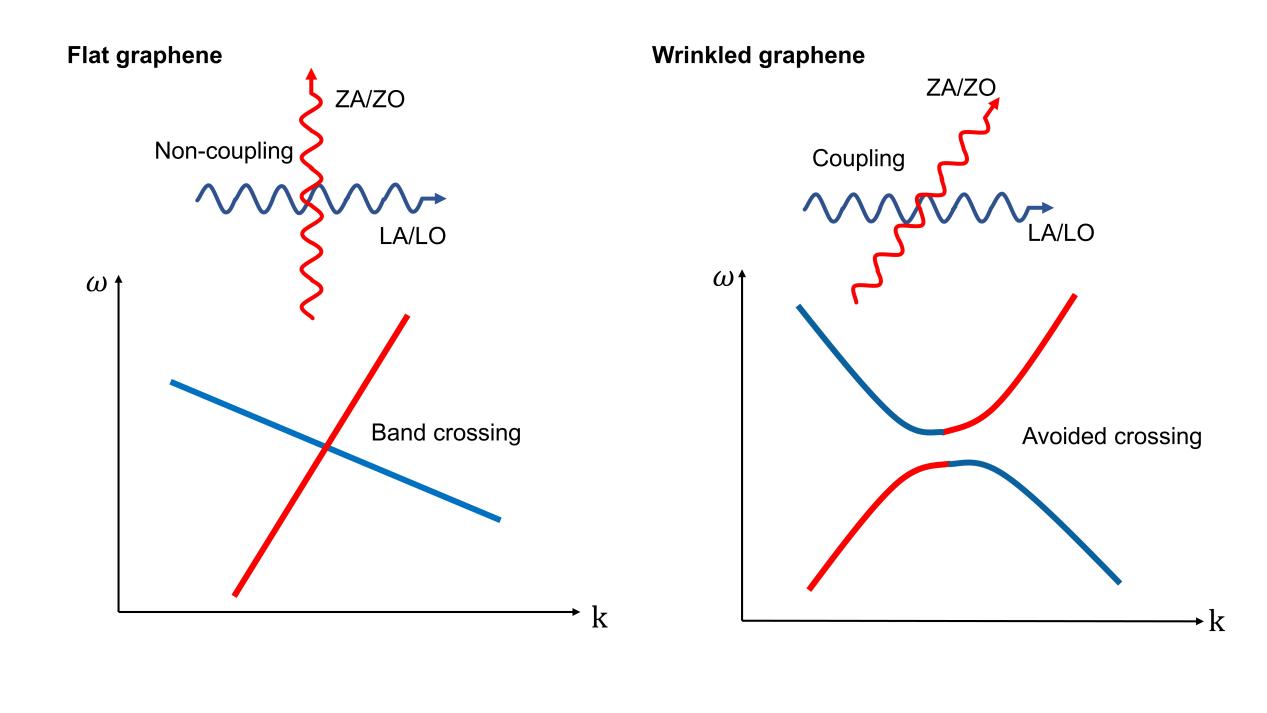


**Figure S14.** In flat graphene, out-of-plane flexural and in-plane longitudinal phonons are orthogonal, so they do not interact and cross directly in the band structure. Wrinkling breaks this orthogonality, allowing coupling between phonons of similar frequency. The resulting hybridized modes exhibit avoided crossings between the corresponding branches.


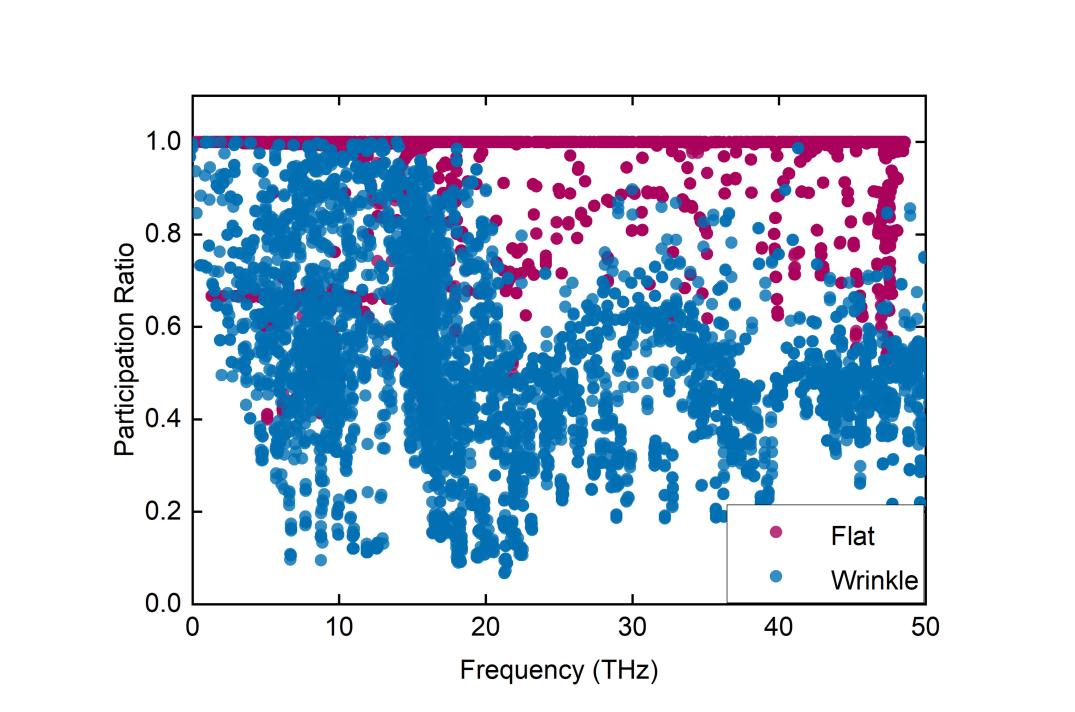


**Figure S15.** Participation ratio as a function of frequency for flat and wrinkled BLG.

**Supplementary Note 5. Electrical measurement**

The BLG samples were supported by SiO2/Si substrate with a 5×5μm squared holes. Gold electrodes were used to energize the samples, the distance between the gold electrode panels is 8μm. The electrical resistances of wrinkled BLG samples were measured by 2-probe method after annealing at 300°C under vacuum. The DC source used for the measurements was ADCMT 6243, two Keithley 2002 digit multimeters were used for voltage and current measurements respectively, and the probe station was Semishare H8.


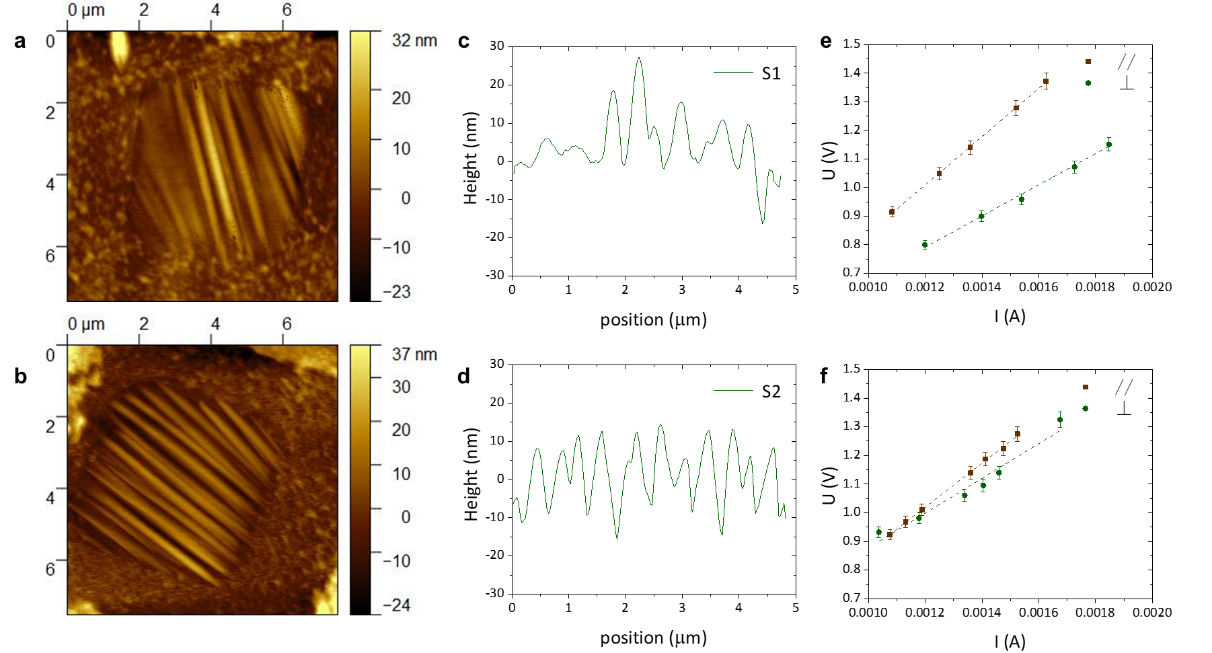


**Figure S16.** AFM scanning images of wrinkled samples (a) S1 and (b) S2, the cross-section profile of (c) S1 and (d) S2, and the current-voltage curves of (e) S1 and (f) S2.

**Table S5** Electrical resistance measurement results of wrinkled BLG

| Sample | *R*_//_ / Ω | *R*_⊥_ / Ω | *R*_//_ /*R*_⊥_ |
| --- | --- | --- | --- |
| S1 | 843±9 | 546±20 | 1.54±0.06 |
| S2 | 768±11 | 606±36 | 1.27±0.08 |

The measurement uncertainty of electrical resistance can be determined by Eq. (10), where *R* is the electrical resistance and the slope of U-I curve, *r^2^* is the R-square of U-I curve, and *n* is the number of measurements. And the uncertainty of *R*_//_ /*R*_⊥_ can be obtained by the law of propagation of uncertainties as shown in Eq. (11).

$\delta\left( R \right)=R\times\sqrt{\frac{\frac{1}{r^{2}}-1}{n-2}}$ (10)

$\delta\left( \frac{R_{//}}{R_{\perp}} \right)=\sqrt{\frac{1}{R_{\perp}^{2}}{\delta\left( R_{//} \right)}^{2}+\frac{R_{//}^{2}}{R_{\perp}^{4}}{\delta\left( R_{\perp} \right)}^{2}}$ (11)
